# Association between Portal Vein Thrombosis and Survival in Non-Liver-Transplant Patients with Liver Cirrhosis: A Systematic Review of the Literature

**DOI:** 10.1155/2015/480842

**Published:** 2015-02-24

**Authors:** Xingshun Qi, Junna Dai, Man Yang, Weirong Ren, Jia Jia, Xiaozhong Guo

**Affiliations:** ^1^Department of Gastroenterology, General Hospital of Shenyang Military Area, Shenyang 110840, China; ^2^Xijing Hospital of Digestive Diseases, Fourth Military Medical University, Xi'an 710032, China; ^3^Department of Gastroenterology, Songgang People's Hospital, Shenzhen 518105, China; ^4^Department of Digestive Diseases, Sanmenxia Central Hospital, Henan University of Science and Technology, Xiaoshan Road, Sanmenxia 472000, China; ^5^Department of Emergency, Shaanxi Provincial People's Hospital, Xi'an 710068, China

## Abstract

A systematic review of the literature was performed to analyze the association between portal vein thrombosis (PVT) and survival in non-liver-transplant patients with liver cirrhosis. PubMed, EMBASE, and Cochrane Library databases were searched for all relevant papers which evaluated the prognostic value of PVT in predicting the survival of liver cirrhosis. Meta-analyses were not conducted because the ways of data expression and lengths of follow-up were heterogeneous among studies. Overall, 13 papers were included. The 5-day, 6-week, and 1-year mortality were investigated in 1, 3, and 1 studies, respectively; and all of them were not significantly different between cirrhotic patient with and without PVT. By comparison, the 3-year mortality was reported in 1 study; and it was significantly increased by the presence of PVT. The overall mortality was analyzed in 5 studies; and the association with overall mortality and PVT was significant in 4 studies, but not in another one. However, as for the cirrhotic patients undergoing surgical or interventional shunts, the overall mortality was not significantly associated with the presence of PVT in 4 studies. In conclusion, the presence of PVT might be associated with the long-term mortality in non-liver-transplant patients with liver cirrhosis, but not with the short-term mortality.

## 1. Introduction

Liver cirrhosis is the end stage of chronic liver diseases resulting in the life-threatening complications [1, 2]. According to the Global Disease Burden Study, it is the 12th cause of death, the 17th cause of years of life lost, and the 23rd cause of disability-adjusted life year in the world [3, 4]. The total number of global deaths attributed to liver cirrhosis is increased from 777,800 (95% uncertainty interval: 663,100–867,900) in 1990 to 1030,800 (95% uncertainty interval: 868,800–1160,500) in 2010 [3]. The total number of global disability-adjusted life years due to liver cirrhosis is also increased from 24,327,000 (95% uncertainty interval: 20,693,000–27,179,000) in 1990 to 31,027,000 (95% uncertainty interval: 25,965,000–34,645,000) in 2010 [4]. The natural history of liver cirrhosis is largely influenced by the occurrence of variceal bleeding, ascites, and infection [5–8]. Child-Pugh score, Model for the End-Stage Liver Disease (MELD) score, and their components (i.e., bilirubin, albumin, prothrombin time or international normalized ratio, creatinine, encephalopathy, and ascites) are considered as the major predictors for the survival of liver cirrhosis [5, 9]. Recent evidence suggests the potential relationship of the survival with the occurrence of portal vein thrombosis (PVT) in liver cirrhosis [10]. However, the conclusions are inconsistent among studies yet. Herein, we have systematically analyzed the literatures regarding the association of PVT with the short- and long-term survival in non-liver-transplant patients with liver cirrhosis.

## 2. Methods

### 2.1. Search Strategy and Study Selection

All papers regarding PVT had been retrieved via the PubMed, EMBASE, and Cochrane Library databases [11]. Among them, the clinical studies with more than 10 patients were identified [12]. The studies that evaluated the association between PVT and survival/death in liver cirrhosis were further identified. The exclusion criteria were as follows: (1) only malignant patients with PVT were enrolled; (2) PVT developed after surgery or interventional treatments; (3) PVT developed in noncirrhotic patients; (4) only LT recipients with PVT were enrolled; (5) no control group was included (i.e., patients without PVT); (6) the survival was not compared between case and control groups; and (7) no separate data regarding PVT was extracted. Notably, the association of PVT with the survival of LT recipients was evaluated in another meta-analysis study. Therefore, the papers including only LT recipients with PVT were excluded from the present systematic review.

### 2.2. Data Extraction

The following data were extracted: first author, publication year, study design, enrollment period, target population, treatment modalities, total number of observed patients, and number/percentage of patients with PVT. Additionally, we collected the data regarding the mortality and/or survival rate in cirrhotic patients with and without PVT. If the original data were not reported, the odds ratios (ORs) with 95% confidence interval (CI), hazard ratios (HRs) with 95% CI, or *P* values were collected to express the difference in the mortality and/or survival rate between the two groups. Data were not synthesized because they were expressed in different ways.

### 2.3. Grade of Evidence

The evidence was classified into high and low grade. The evidence was of high grade, if any one of the 2 following points was met: (1) a multivariate analysis was performed to explore the statistically significant difference; (2) if only a univariate analysis was performed, the baseline Child-Pugh class or MELD score should be matched between patients with and without PVT. Otherwise, the evidence was of low grade.

## 3. Results

### 3.1. Characteristics of Included Studies

**Figure 1:**
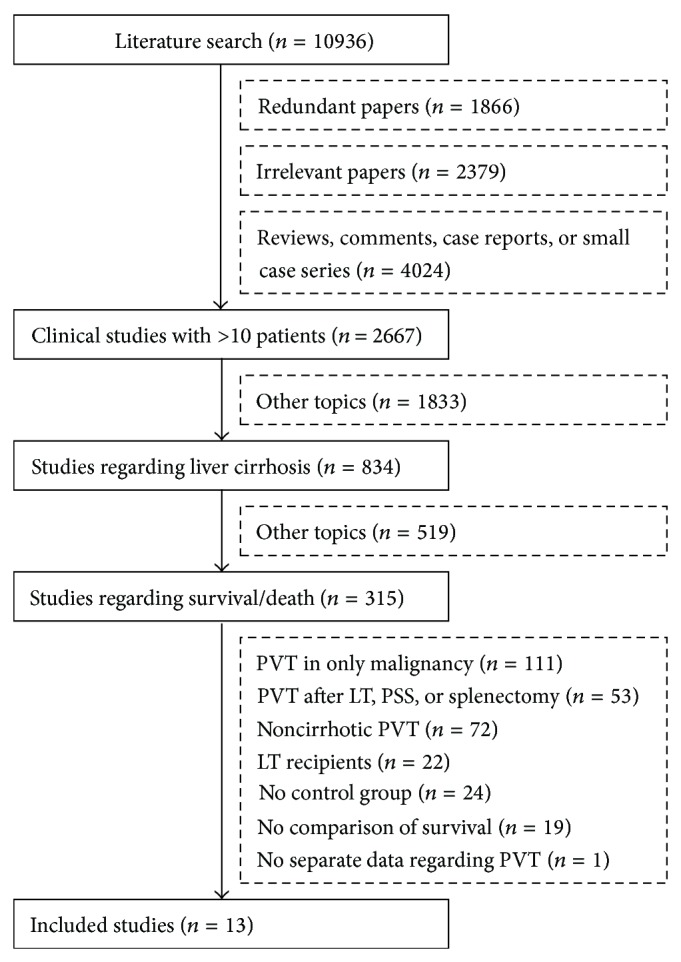
Flowchart of study selection.

Initially, 10936 papers regarding PVT were identified. Among them, 13 papers were eligible for this systematic review [13–25] (Figure 1). The characteristics of included studies were summarized in Table 1. According to the regions, 4 studies were performed in China Taiwan, 3 studies in Italy, 2 studies in USA, 1 study in Canada, 1 study in France, 1 study in Portugal, and 1 study in UK. According to the enrollment periods, 3 studies were launched before 1990 [21, 23, 24], 3 studies between 1990 and 2000 [16, 22, 25], and 6 studies after 2000 [13–15, 17, 19, 20]. The information regarding the enrollment periods was not available in 1 study [18]. According to the publication forms, 3 studies were published in abstracts, and 10 studies in full-texts. Hepatocellular carcinoma was excluded in 4 studies [14, 17, 18, 22], but not in 6 studies [13, 15, 16, 19–21, 25]. The information regarding the exclusion of hepatocellular carcinoma was not available in 3 studies [21, 23, 24]. The prevalence of PVT in liver cirrhosis was 7%–25%.

Data were expressed in different ways (Table 2). Multivariate analyses were performed in 7 studies [13–16, 18, 19, 25], and only univariate analyses were performed in 6 studies [17, 20–24]. Of these studies without multivariate analyses, 2 had similar proportions of Child-Pugh classes between patients with and without PVT [17, 22], 2 had significantly different proportions of Child-Pugh classes between the two groups [21, 23], and 2 did not clearly report any relevant information [20, 24]. Thus, 9 studies were considered to have relatively high-grade evidence.

### 3.2. 5-Day Mortality

Amitrano and colleagues found that the proportion of PVT was not significantly different between cirrhotic patients who died within 5 days after acute variceal bleeding and those who did not (5/27 versus 27/158, *P* = 0.323) [13].

### 3.3. 6-Week Mortality

Chen and colleagues reported that PVT was significantly associated with an increased 6-week mortality in the univariate Cox regression analysis (HR = 3.19, 95% CI = 1.59–6.41, *P* = 0.001) [15]. But it was not identified as the independent predictor for the 6-week mortality in the multivariate Cox regression analysis.

D'Amico and colleagues did not find PVT as a significant predicator for the 6-week mortality in the multivariate logistic regression analysis [16].

Lee and colleagues showed that the proportion of PVT was not significantly different between cirrhotic patients who died within 6 weeks after the cessation of initial esophageal variceal bleeding than in those who did not (40% [4/10] versus 17.2% [15/87], *P* = 0.102) [20].

### 3.4. 1-Year Mortality

Ferreira and colleagues found that the 1-year mortality was not significantly different between cirrhotic patients with and without PVT (the data was not shown) [18].

### 3.5. 3-Year Mortality

Ferreira and colleagues found that the 3-year mortality was significantly associated with PVT in cirrhotic patients (*P* = 0.001) [18]. The prognostic significance of PVT for the 3-year mortality was confirmed in the multivariate logistic regression analysis (OR = 6, 95% CI = 2–18).

### 3.6. Overall Mortality

In a prospective longitudinal study by Attili and colleagues, the overall mortality was significantly higher in patients who developed PVT during follow-up than in those who did not (60% [15/25] versus 9.6% [10/104]) [14]. In the Kaplan-Meier curve, the cumulative survival rate was significantly lower in patients with incident PVT than in those without (*P* < 0.00001, by log-rank test). The prognostic significance of incident PVT was also confirmed in the multivariate Cox regression analysis.

In a case-control study by Ferreira et al., the Kaplan-Meier curve demonstrated a significantly poorer long-term survival in PVT patients (*P* = 0.034) [18]. The association between PVT and mortality was significant in patients with Child-Pugh classes A and B, but not in those with Child-Pugh class C.

In a randomized controlled trial by Hung and colleagues, the univariate Cox regression analysis demonstrated that PVT was significantly associated with a poorer overall survival in cirrhotic patients with gastric variceal bleeding undergoing the secondary prevention (HR = 6.024, 95% CI = 2.770–13.158, and *P* < 0.001) [19]. In the multivariate Cox regression analysis, PVT remained the independent predictor for the overall survival (HR = 3.390, 95% CI = 1.499–7.692, and *P* = 0.003).

In a retrospective study by Wu and colleagues, the univariate Cox regression analysis demonstrated that PVT was significantly associated with the survival in cirrhotic patients with gastric variceal bleeding after endoscopic therapy (HR = 12.6, 95% CI = 5.93–26.72, and *P* < 0.01) [25]. The statistical significance was confirmed in the multivariate Cox regression analysis (HR = 6.99, 95% CI = 2.42–20.16, and *P* < 0.01).

Only one study by Doumit and colleagues found a similar incidence of death between cirrhotic patients with and without PVT (16% versus 21%) [17].

### 3.7. Mortality after Surgical or Interventional Portosystemic Shunt

In a study by Windle and Peacock, the overall mortality after end-to-side anastomosis of the portal vein to the inferior vena cava was not significantly different between cirrhotic patients with and without PVT (7/10 [70%] versus 25/47 [53%], *P* = 0.138, by Chi-square test) [24].

In a study by Sarfeh, the overall mortality after portal decompressive procedures, such as portocaval or mesocaval shunt, was not significantly different between patients with PVT and patent portal veins (10/18 [56%] versus 26/68 [38%], *P* = 0.185, by Chi-square test) [23].

In a study by Orloff and colleagues, the Kaplan-Meier curve demonstrated a similar survival rate between patients with and without PVT after emergency (30 days: 69% versus 73%, 1 year: 66% versus 65%, 5 years: 65% versus 61%, 10 years: 55% versus 52%, and 15 years: 51% versus 45%) or elective (30 days: 95% versus 98.6%, 1 year: 90% versus 95%, 5 years: 70% versus 71%, 10 years: 65% versus 65%, and 15 years: 60% versus 61%) surgical portocaval shunt [21].

In a study by Perarnau and colleagues, the Kaplan-Meier curve demonstrated that the probability of survival after transjugular intrahepatic portosystemic shunt (TIPS) was not significantly different between patients with and without PVT (1 year: 80% versus 84%, 2 years: 72% versus 70%, and 4 years: 55% versus 52%; *P* = 0.58, by log-rank test) [22]. Additionally, the median survival time was 50 ± 12 months and 36 ± 5 months in patients with and without PVT, respectively.

## 4. Discussion

Our study was the first to systematically analyze the effect of PVT on the survival of non-liver-transplant patients with liver cirrhosis. The majority of papers included in our systematic review provided the high-grade evidence. The major findings included the following: (1) PVT might not be significantly associated with the 5-day, 6-week, and 1-year mortality of liver cirrhosis; (2) PVT might be significantly associated with an increased 3-year mortality; (3) PVT might increase the overall mortality during follow-up; and (4) a preexistent PVT did not impact the survival of cirrhotic patients treated with surgical or interventional shunt.

It appears that the short-term survival is not influenced by the presence of PVT. By comparison, the markers of liver dysfunction play a more important role in the prediction of the short-term survival. For example, Amitrano et al. found that Child-Pugh and MELD scores were two significant factors associated with the 5-day mortality [13]; D'Amico and De Franchis also suggested that an increased bilirubin level, a decreased albumin, and encephalopathy predicted a higher 6-week mortality [16]; and Chen et al. reported that MELD score was the independent predictor for the 6-week mortality [15]. Thus, the prognostic value of PVT might be masked by the progressive deterioration of liver function during a relatively short follow-up. In addition, the presence of advanced HCC was often considered the independent predictor for the short-term mortality [13, 15, 16].

The long-term survival is negatively influenced by the presence of PVT in 4 of 5 studies [14, 18, 19, 25], which indicates the negative impact of PVT on the survival [26]. On the contrary, this is not supported by one study that was published in the abstract form [17]. However, the results of this study should be cautiously interpreted because nearly all decompensation events occurred more frequently in PVT group than in non-PVT group (ascites: 41% versus 28%; variceal bleeding: 39% versus 20%; spontaneous bacterial peritonitis: 23% versus 9%; hepatocellular carcinoma: 20% versus 8%; and portosystemic encephalopathy: 32% versus 27%), but the mortality was relatively lower (16% versus 21%) [17].

Villa and colleagues conducted a randomized controlled trial to explore the role of anticoagulation for the primary prevention of PVT in liver cirrhosis [27]. The investigators found that the prophylactic anticoagulation could not only decrease the incidence of PVT, but also reduce the development of hepatic decompensation events and improve the survival. Recently, it has been also proposed that the identification of thrombotic risk factors for PVT should be helpful to stratify the benefits of prophylactic anticoagulation in liver cirrhosis [28, 29]. However, we have to acknowledge that few thrombotic risk factors for PVT have been clearly established in a small number of patients with liver cirrhosis [30–33]. In addition, anticoagulation could be effective for the treatment of PVT in liver cirrhosis [34–37]. Thus, anticoagulation should be recommended for the management of PVT in liver cirrhosis. Importantly, if a PVT could be avoided in the future or a previously thrombosed portal vein was successfully recanalized, the survival might be further improved.

On the other hand, TIPS is also regarded as a treatment of choice for PVT in liver cirrhosis [38]. Its advantages are that the occluded vessels can be easily recanalized by endovascular techniques, and the patency of vessels can be effectively maintained by an accelerated blood flow through the main portal vein and shunt. However, its primary treatment goal remains to resolve the portal hypertension-related complications [39] because no controlled trials are available to confirm the benefits of TIPS versus anticoagulation for recanalizing the thrombosed portal vein [40]. Despite that, our systematic review showed that the survival of cirrhotic patients who were treated with TIPS or surgical shunts was not influenced by the presence of a preexistent PVT. The indirect evidence suggested that the creation of a portosystemic shunt might improve the survival of liver cirrhosis with PVT.

Our study had several limitations. First, we could not combine the data reported by these included studies into a general result. This was primarily because the ways of data expression and lengths of follow-up were heterogeneous among studies. For example, some papers reported the total number of patients and the number of death events; by contrast, the others reported the ORs or HRs. Additionally, some papers just reported the short-term survival; contrarily, the others reported the survival during the total follow-up. Second, we could not extract the information regarding whether or not the degree of PVT (complete occlusion versus partial occlusion) and Child-Pugh classes (class C versus classes A/B) were able to further stratify the effect of PVT on the survival of liver cirrhosis. Third, some included papers did not exclude the cases with hepatocellular carcinoma. This precluded us from distinguishing whether the nature of PVT was benign or malignant. The presence of malignant PVT was associated with very poor survival. Fourth, although the search strategy was extensive, the number of relevant papers was relatively small. Thus, the reproducibility of these findings remained to be confirmed.

In conclusion, the presence of PVT might be negatively associated with the long-term survival, but not with the short-term survival, which might support the necessity of anticoagulation or other treatment modalities for maintaining the portal vein patency in liver cirrhosis. Certainly, we should acknowledge that the effect of PVT on the long-term survival after surgical shunt or TIPS was not significant.

## Figures and Tables

**Table 1:** Characteristics of included studies.

First author (year)	Country	Design	Period	Target population	Number of total patients	PVT number (%)
Amitrano (2012) [13]	Italy	Prospective cohort study (full-text)	2010.1–2011.7	LC with acute EVB vasoactive therapy, antibiotics, and EVL	185	32 (17%)

Attili (2012) [14]	Italy	Prospective longitudinal study (abstract)	2000.2–2005.7	LC without HCC	129	25 (19%)

Chen (2012) [15]	Taiwan	Retrospective study (full-text)	2005.7–2009.12	LC with acute EVB treated with somatostatin, antibiotics, and EVL	101	25 (25%)

D'Amico (2003) [16]	Italy	Multicenter, prospective, cohort study (full-text)	1997.10–1998.1^*^	LC with hematemesis and/or melena (treatments include vasoactive drugs, endoscopic therapy, combination of endoscopic and vasoactive therapy, balloon tamponade alone, or none)	291^*^	37 (13%)^*^

Doumit (2009) [17]	Canada	NA (abstract)	2002	LC who underwent portal vein Doppler US and who had no preexisting HCC, TIPS, or surgical shunt in situ or prior to LT	398	44 (11%)

Ferreira (2010) [18]	Portugal	Case-control study (abstract)	NA	*Case group:* consecutive LC with non-HCC PVT; *control group:* decompensated LC matched for Child-Pugh and MELD scores, age, and etiology of LC	*Case group: n* = 40; *Control group: n* = 135	

Hung (2012) [19]	Taiwan	RCT (full-text)	2007.4–2011.3	LC with acute GVB after primary hemostasis using gastric variceal obturation therapy (randomized to repeated gastric variceal obturation alone or in combination with nonselective *β*-blockers)	95	13 (14%)

Lee (2010) [20]	Taiwan	Retrospective study (full-text)	2005.12–2008.2	LC after the cessation of acute EVB	97	19 (20%)

Orloff (1997) [21]	USA	Prospective cohort study (full-text)	1958–1991	LC with acutely bleeding esophagogastric varices or a previous episode of bleeding esophagogastric varices treated with emergency or elective portacaval shunt	1300	85 (7%)

Perarnau (2010) [22]	France	Retrospective study (full-text)	1990–2004	LC undergoing TIPS for emergency bleeding hemostasis or rebleeding prevention	273	27 (10%)

Sarfeh (1979) [23]	USA	Retrospective study (full-text)	1972–1978	Biopsy-proved LC treated with portal decompression surgery for active or previous variceal hemorrhage	86	18 (21%)

Windle (1975) [24]	UK	Retrospective study (full-text)	1950–1957	Patients who underwent end-to-side anastomosis of the portal vein to the inferior vena cava for portal hypertension secondary to intrahepatic disease	57	10 (17.5%)

Wu (2002) [25]	Taiwan	Retrospective study (full-text)	1992.11–1998.10	LC with acute GVB treated with endoscopic N-butyl-2-cyanoacrylate injection	83	15 (18%)

EVB: esophageal variceal bleeding; EVL: endoscopic variceal ligation; GVB: gastric variceal bleeding; HCC: hepatocellular carcinoma; LC: liver cirrhosis; MELD: model for end-stage liver diseases; NA: not available; PVT: portal vein thrombosis; RCT: randomized controlled trial; TIPS: transjugular intrahepatic portosystemic shunt.

Notes: ^*^the data in the training set.

**Table 2:** An overview of survival data expression.

Author (year)	Data expression							
	Number and/or percentage of death events	Survival time	Kaplan-Meier curve	Log-rank test	Univariate logistic regression analysis	Multivariate logistic regression analysis	Univariate Cox regression analysis	Multivariate Cox regression analysis
Amitrano (2012) [13]	***√***	**×**	**×**	**×**	***√***	***√***	**×**	**×**
Attili (2012) [14]	***√***	**×**	***√***	***√***	**×**	**×**	**×**	**×**
Chen (2012) [15]	**×**	**×**	**×**	**×**	**×**	**×**	***√***	***√***
D'Amico (2003) [16]	**×**	**×**	**×**	**×**	***√***	***√***	**×**	**×**
Doumit (2009) [17]	***√***	**×**	**×**	**×**	**×**	**×**	**×**	**×**
Ferreira (2010) [18]	**×**	**×**	**×**	***√***	***√***	***√***	**×**	**×**
Hung (2012) [19]	**×**	**×**	**×**	**×**	**×**	**×**	***√***	***√***
Lee (2010) [20]	***√***	**×**	**×**	**×**	**×**	**×**	**×**	**×**
Orloff (1997) [21]	***√***	**×**	***√***	**×**	**×**	**×**	**×**	**×**
Perarnau (2010) [22]	***√***	***√***	***√***	***√***	**×**	**×**	**×**	**×**
Sarfeh (1979) [23]	***√***	**×**	**×**	**×**	**×**	**×**	**×**	**×**
Windle (1975) [24]	***√***	**×**	**×**	**×**	**×**	**×**	**×**	**×**
Wu (2002) [25]	**×**	**×**	**×**	**×**	**×**	**×**	***√***	***√***
